# Modeling Social Transmission Dynamics of Unhealthy Behaviors for Evaluating Prevention and Treatment Interventions on Childhood Obesity

**DOI:** 10.1371/journal.pone.0082887

**Published:** 2013-12-17

**Authors:** Leah M. Frerichs, Ozgur M. Araz, Terry T. – K. Huang

**Affiliations:** College of Public Health, University of Nebraska Medical Center, Omaha, Nebraska, United States of America; University of Catania, Italy

## Abstract

Research evidence indicates that obesity has spread through social networks, but lever points for interventions based on overlapping networks are not well studied. The objective of our research was to construct and parameterize a system dynamics model of the social transmission of behaviors through adult and youth influence in order to explore hypotheses and identify plausible lever points for future childhood obesity intervention research. Our objectives were: (1) to assess the sensitivity of childhood overweight and obesity prevalence to peer and adult social transmission rates, and (2) to test the effect of combinations of prevention and treatment interventions on the prevalence of childhood overweight and obesity. To address the first objective, we conducted two-way sensitivity analyses of adult-to-child and child-to-child social transmission in relation to childhood overweight and obesity prevalence. For the second objective, alternative combinations of prevention and treatment interventions were tested by varying model parameters of social transmission and weight loss behavior rates. Our results indicated child overweight and obesity prevalence might be slightly more sensitive to the same relative change in the adult-to-child compared to the child-to-child social transmission rate. In our simulations, alternatives with treatment alone, compared to prevention alone, reduced the prevalence of childhood overweight and obesity more after 10 years (1.2–1.8% and 0.2–1.0% greater reduction when targeted at children and adults respectively). Also, as the impact of adult interventions on children was increased, the rank of six alternatives that included adults became better (i.e., resulting in lower 10 year childhood overweight and obesity prevalence) than alternatives that only involved children. The findings imply that social transmission dynamics should be considered when designing both prevention and treatment intervention approaches. Finally, targeting adults may be more efficient, and research should strengthen and expand adult-focused interventions that have a high residual impact on children.

## Introduction

The worldwide growth in overweight and obesity has created negative health, social and economic consequences for children, adults, and society as a whole [1]–[3]. In the US, alongside increasing adult overweight and obesity rates, the problem has grown among children [4], [5]. Some research indicates increases in US childhood overweight and obesity rates may be slowing [5], but we still need strategies to accelerate a downward trend in order to abate forthcoming obesity-related health and economic consequences [6]. Research that improves our understanding of the complex dynamics of social spread of obesity among children via both peer and adult influences may help identify key leverage points, and guide resource allocation to the most impactful combination of intervention strategies.

The immediate cause of overweight and obesity is energy imbalance, but complex interactions of multi-level factors including individual human biology, behavior, and environment give rise to the current worldwide epidemic [7]. Christakis and Fowler [8] found evidence that the adult obesity epidemic appears to be spreading through social ties, based on the clustering of surveyed individuals according to their BMIs and increased chance of becoming obese based on different ties. Social ties may transfer obesity and obesity-related behaviors through pathways of social norms, capital (i.e., resources, information and people accessible through a social network), and stress [9].

Additional evidence strengthens the role of social influence in both adult and child populations. Research continually uncovers adult-to-adult [8], adult-to-child [10]–[14], and child-to-child [14]–[18] associations and influence in terms of obesity and obesity-related attitudes, norms, and behaviors (i.e., nutrition and physical activity). Furthermore, a few recent obesity interventions found that targeting parents only may have a significant residual impact on children in regards to behavior and weight change [19]–[21].

The interdependencies among parent and peer influences on childhood obesity are difficult to understand using linear models. System dynamics modeling can help explore the complex multi-level social influences on child obesity risks, and identify potential research gaps and plausible intervention levers with policy implications by analyzing outcome patterns [22]. For example, system dynamics provides a methodology to test combinations of prevention and treatment intervention impact directed towards adults and children on childhood overweight and obesity trends, which can enhance our ability to understand the combination of strategies with potential for greatest impact.

### Obesity and Complex Systems Modeling Background

Prior research has applied complex systems modeling to study obesity dynamics [23]–[29]. For our research we build upon several models that use mathematical and system dynamics methodologies to consider excess weight as a consequence of the transmission of unhealthy lifestyles from one individual to another [26], [29]–[31]; however, to our knowledge no models have simultaneously accounted for peer and adult transmission of behaviors for childhood overweight and obesity.

Computational and quantitative models of obesity have been used to understand the dynamics of energy regulation at the individual and biological level in order to understand issues such as weight cycling [23], [24], [32]–[34]. Research via system dynamics modeling has built upon these energy regulation models in order to capture these dynamics throughout the life course and simulate future population level trends [25], [28]. These models were also used to test and formulate information about policy interventions, but they do not explicitly account for the social transmission of obesogenic behaviors.

Karanfil et al's [27] agent-based framework provided value for understanding how opinions about nutrition and physical activity can be transferred through social ties. Other researchers have used mathematical models to understand the growth of obesity via social transmission [26], [29]–[31]. Evangelista et al [30] used peer pressure to become a fast food eater as a parameter in a model to simulate changes in overweight and obesity rates. Researchers in Spain built models with similar social transmission parameters to simulate population obesity rate growth for different age groups, including infants and adults, and used the models to test the impact of different combinations of prevention and treatment interventions [26], [29], [31]. Unfortunately, these models do not provide the functionality to understand different levels of influence on children from their peers and adults.

The aim of our research is to gain insight into potential research gaps and plausible levers for future childhood obesity prevention and treatment intervention and policy research. We hypothesize that the multi-level and dissimilar quantifications of social transmission of overweight and obesity from adult-to-adult, adult-to-child, and child-to-child create different patterns of overweight and obesity. In our research, we construct and parameterize a system dynamics model of the social transmission of behaviors that cause childhood overweight and obesity through adult and peer influence. Our objectives are: (1) to assess the sensitivity of childhood overweight and obesity prevalence to peer and adult social transmission rates, and (2) to test combinations of prevention and treatment interventions, with varying degrees of adult intervention impact on children and vice versa, on the prevalence of childhood overweight and obesity.

## Methods

We used Vensim PLE (Ventana Systems, Inc., Harvard, MA) to build and simulate the model and test the dynamic hypotheses and assumptions. We conducted two-way sensitivity analyses on social transmission rates from adult-to-child and from child-to-child. Alternative combinations of prevention and treatment interventions were tested by varying model parameters of social transmission and rates of overweight and obese individuals engaged in weight loss behaviors. We designed an experiment to explore the alternative combinations' impacts on childhood overweight and obesity prevalence using a set of scenarios, each with varying adult intervention impact on children and vice versa.

### Model Description

**Figure 1:**
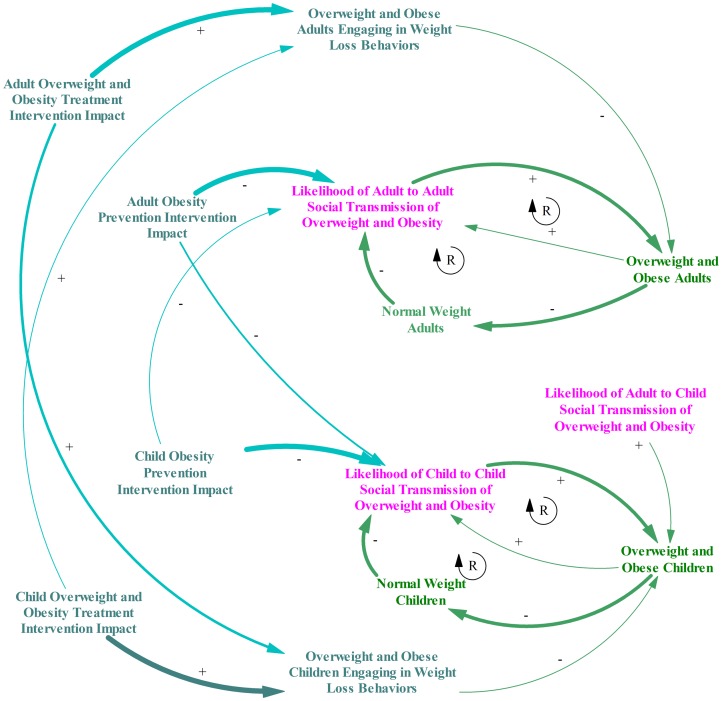
Causal Loop Diagram of Adult and Child Social Transmission of Obesity. This figure shows a causal loop diagram that illustrates the elements of the system we used for our research to model and test hypotheses regarding child and adult social transmission of unhealthy behaviors causing overweight and obesity. Adult level elements are shown in green and child level elements are shown in pink. Each element in the system is included with arrows drawn between elements to indicate relationships where they exist. The arrows are labeled with plus signs if a positive relationship exists between the elements and minus sign if an inverse relationship exists. The diagram includes adult-to-adult, adult-to-child, and child-to-child social transmission elements with arrows indicating how each increases overweight and obese individuals in the population for each respective age group. The overweight and obese individuals for each age group are shown with arrows to indicate its positive relationship with social transmission and inverse relationship with normal weight individuals. Finally normal weight individuals in each age group have arrows indicating an inverse relationship to social transmission of overweight and obesity. Within the elements and arrows described, circular arrows with a capital “R” are shown in the center to indicate reinforcing feedback loops. Elements of intervention impact are also included with arrows and plus/minus signs indicating relationships. Treatment intervention impact for children and adults are shown with negative labeled arrows to overweight and obese children and adults actively engaging in weight loss behaviors. Prevention intervention impact for children and adult levels are shown with negatively labeled arrows to social transmission. Intervention impact lines are shown at different widths to indicate differences in relative magnitude of impact. The thickest lines are shown regarding adult-to-adult impact and child-to-child impacts. Lines of medium thickness are shown regarding adult-to-child impact. Finally the thinnest lines are shown regarding child to adult impact.

A causal loop diagram illustrates the elements of the system we used to model and test hypotheses regarding child and adult social transmission of unhealthy behaviors causing overweight and obesity (Figure 1).

#### Model Boundaries

The model boundary for our research included social transference of unhealthy behaviors at adult-to-adult, adult-to-child, and child-to-child levels. The transmission was assumed to occur through social influences on food consumption and physical activity behaviors via norms, attitudes, behaviors, and provision of interpersonal material and physical structures and resources. Previous similar mathematical models have used a broad interpretation of social encounters and have assumed factors of genetics and environment to be embedded within this social transmission factor [29]. For our research we considered these outside the system boundaries.

#### Model Elements

Figure 1 shows the elements of the system, which include an individual's health status related to weight (i.e., normal weight, overweight, and obese adults and similarly normal weight, overweight, and obese children). The levels of each of these elements influence the social transmission of overweight and obesity: adult levels influence adult-to-adult and adult-to-child transmission, and child levels influence child-to-child. We assumed child-to-adult transmission of these unhealthy behaviors was negligible.

Several elements were included in modeling intervention impact (Figure 1). Treatment intervention increases the level of overweight and obese children and adults actively engaging in dieting and physical activity to lose weight. Prevention intervention decreases the social transmission of obesity-related unhealthy behaviors. The obesity intervention influences both the targeted age group (e.g., adults) and opposite age group (e.g., children) based on the assumption that adults and children will model intervention-induced healthy behavior change for others. Rather than attempting to change individual behaviors only, obesity interventions may target psychosocial variables in order to encourage the intervention participant to actively model and encourage healthy behaviors among their social contacts. For example, a family centered model which was developed for addressing obesity would potentially include how parents may influence children through mechanisms of modeling, parenting practices of reinforcement and encouragement, and changes to the home environment [35]. Thus the model includes an explicit intervention impact parameter (apart from adult-to-child and child-to-child social transmission) to capture the potential to actively engage targeted individuals to model and encourage healthy behaviors among the other age group at varying degrees. However, the impact on the non-targeted age group is of a lesser magnitude. The line weights in Figure 1 indicate relative differences among the impact's magnitude.

#### Feedback Loops

Increased numbers of overweight and obese individuals raise the likelihood of social transmission of peer-to-peer unhealthy behaviors (i.e., greater contact of normal weight with overweight and obese individuals), which in turn increases the number of overweight and obese individuals. Additionally, increased numbers of overweight and obese individuals in a fixed population will decrease numbers of normal weight individuals, which also raises the likelihood of social transmission of peer-to-peer unhealthy behaviors (see Figure 1). Thus there are two reinforcing loops seen in both the adult and child populations: (1) a loop between the increase in overweight and obesity that leads to a rise in the likelihood of social transmission, and (2) a loop from the increase in overweight and obesity that leads to a decrease in normal weight population, which leads to a subsequent increase in the likelihood of maintained social transmission.

### Mathematical Model

**Figure 2:**
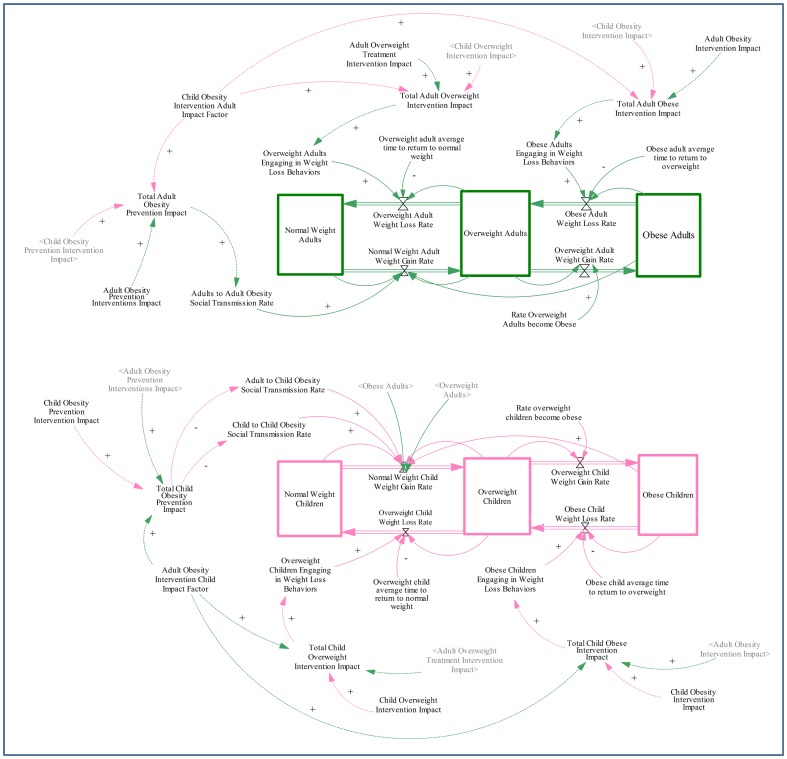
Stock Flow Diagram of Adult and Child Social Transmission of Obesity. This figure shows the stock flow diagram built in Vensim. Adult level influences are shown in green and child level influences in pink. The core model elements are shown in solid lines, and intervention variables are indicated in dotted lines. Stocks of normal weight, overweight and obese adults are shown in green and stocks of normal weight, overweight, and obese children are shown in pink. Variables are shown with arrows to the flow equation they are included in. For example, child-to-child and adult-to-child social transmission rates are included in the flow equation from normal weight children to overweight children stock. Intervention impact variables are also shown with arrows to the behavioral variable they impact. For example, the total child prevention intervention impact reduces the child-to-child and adult-to-child social transmission rates. Finally the adult intervention impact and child intervention impact factors are also indicated and arrows indicate the total intervention impact levels they influence.

We built a stock and flow diagram to model the underlying structure, and governing equations of the model were adapted from previous models [26], [29]–[31]. The stock and flow diagram indicates stocks of normal, overweight, and obese adults and children respectively with flows in-between (Figure 2). In this section, we formulate the mathematical equations used to model the spread of obesity through multi-level transmission of obesity-related behaviors through the social environment.

To build the equations for the model, we made the following assumptions:

- We assume homogenous population mixing for behavioral transmission.
- We assume that unhealthy eating behaviors and low physical activity levels of individuals in the model increase the individual weight for both adults and children.
  - Normal weight adults and children will become overweight over time because overweight and obese contacts transmit their unhealthy behaviors through social contacts (i.e., social transmission rates). Social contact is modeled proportionally to the number of contacts of normal weight with overweight and obese individuals. For children, this transmission is both in terms of proportional contacts with adults as well as their peers. For adults, the transmission occurs only through adult contacts, and children are assumed not to transmit unhealthy behaviors.
  - Overweight adults and children are assumed to become obese proportionally to the total number of overweight adults and children over time.
  - It is assumed that obese and overweight adults and children have potential to adopt behaviors that will lead to weight loss (i.e., diet and physical activity) if conditions and interventions are adequate. Obese and overweight adults and children can transition to overweight and normal weight, respectively, at a rate proportional to the respective stock, based on the extent of the subpopulation that engages in weight loss behaviors.

The model also includes several variables related to the potential for obesity prevention and treatment impact. The following assumptions were made for this purpose:

- Interventions are assumed to impact targeted behavioral variables by either increasing or decreasing them over time linearly.
  - Obesity prevention intervention is assumed to fortify normal weight individuals against transmission of unhealthy behaviors of overweight and obese individuals. Thus prevention intervention impact is assumed to be a decrease to social transmission rates.
  - Overweight and obesity intervention is assumed to help additional individuals in targeted subgroups to engage in weight loss behaviors. Thus treatment intervention impact is assumed to increase the rate of those engaging in weight loss behaviors.
  - Adult obesity prevention intervention is assumed to decrease the adult-to-adult social transmission rate, and child obesity prevention intervention is assumed to decrease both child-to-child and adult-to-child transmission rates (i.e., children are fortified against social transmission from both peers and adults). However, the adult-to-child social transmission rate is assumed to decrease at a discounted rate of the child-to-child transmission rate given children's limited ability to change parental control around issues such as provision of healthy foods [36].
- Interventions are assumed to impact targeted subgroups as well as parallel subgroups with similar behaviors. Thus, adult intervention impact is assumed to also have an influence on child intervention impact and vice versa.
  - The intervention impact on parallel subgroups is assumed to act through mechanisms that are different from social transmission rates alone due to the potential for interventions to actively engage targeted individuals to support and encourage others in their social environment in healthy behaviors.
  - The subgroup impact is assumed to occur proportionally to the direct influence on the targeted subpopulation, modeled via an impact factor. For example, the total adult prevention intervention impact is assumed to be a function of both adult prevention intervention impact and a proportion of the child obesity prevention intervention impact. Similarly, the child obesity prevention intervention impact is assumed to be a function of both the child obesity prevention intervention impact and a proportion of the adult obesity prevention intervention impact. The same relationships are assumed for the overweight and obese child and adult intervention impacts as well.
  - Regardless of prevention or treatment, for the impact of adults on children and vice versa, the impact factor is assumed to be the same.

#### Stock Variables

For the model, child and adult populations were each divided into three subpopulations of normal weight, overweight, and obese.

N_A_, Normal weight adults, individuals with BMI<25 kg/m^2^

S_A_, Overweight adults, individuals with BMI≥25 and<30 kg/m^2^

O_A_, Obese adults, individuals with BMI≥30 kg/m^2^

N_c_, Normal weight children, children<85th percentile on BMI-for age growth charts

S_c_, Overweight children, children between 85^th^ to 95^th^ percentile on BMI-for age growth charts

O_c_, Obese children, children≥95^th^ percentile on BMI-for age growth charts

#### Behavioral Variables

For this model, we included variables of behaviors related to overweight and obesity, including those of diet and physical activity. We also included social transmission rates.

ρ_AWL_ = proportion of overweight adults engaging in weight loss behaviors

ρ_CWL_ = proportion of overweight children engaging in weight loss behaviors

ε_AWL_ = proportion of obese adults engaging in weight loss behaviors

ε_CWL_ = proportion of obese children engaging in weight loss behaviors

*p_SA_* = average time needed for overweight adult to return to normal weight

*p_SC_* = average time needed for overweight child to return to normal weight

*p_OA_* = average time needed for obese adult to return to overweight

*p_OC_* = average time needed for obese child to return to overweight

*γ_A_* = rate at which overweight adults become obese

*γ_C_* = rate at which overweight children become obese

β_AA_ = adult-to-adult social transmission rate

β_CC_ = child-to-child social transmission rate

β_AC_ = adult-to-child social transmission rate

#### Transition Equations

Given the assumptions, the transitions from one state to another are described by the following differential equations of (1)–(6) with the initial conditions of N_A_(0) = N_A0_, S_A_(0) = S_A0_, O_A_(0) = O_A0_, N_C_(0) = N_C 0_, S_C_(0) = S_C 0_, O_C_(0) = O_C 0_.

Normal Weight Adult Stock 
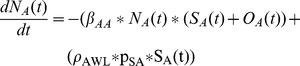
(1)


Overweight Adult Stock 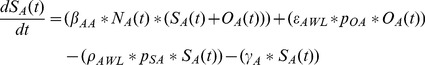
(2)


Obese Adult Stock 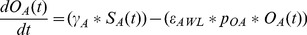
(3)


Normal Weight Children Stock 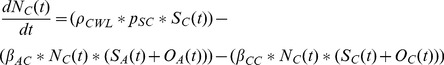
(4)


Overweight Children Stock 
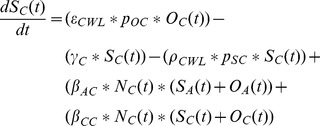
(5)


Obese Children Stock 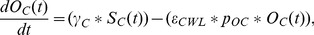
(6)


#### Intervention Variables

For this model, we included variables of intervention impact for each potential subgroup that could be targeted.

η_oc_, childhood obesity treatment intervention impact

η_sc_, childhood overweight treatment intervention impact

η_NC_, childhood prevention treatment intervention impact

η_OA_, adult obesity treatment intervention impact

η_SA_, adult overweight treatment intervention impact

η_NA_, adult obesity prevention treatment intervention impact

ψ_A_, adult obesity intervention impact factor (impact of adult interventions on children)

ψ_c_, child obesity intervention impact factor (impact of child interventions on adults)

, discount factor (accounts for resistance of adult-to-child social transmission to respond to child prevention interventions)

### Intervention Equations

Total childhood obesity prevention intervention impact
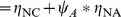
(7)


Total childhood overweight treatment intervention impact 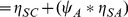
(8)


Total childhood obesity treatment intervention impact 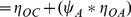
(9)


Total adult obesity prevention intervention impact 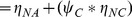
(10)


Total adult overweight treatment intervention impact 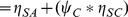
(11)


Total adult obesity treatment intervention impact 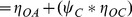
(12)


### Behavioral Impact Equations

Given the assumptions, the impact of interventions on targeted behavioral-related variables can be described by the following equations of (13)–(19). 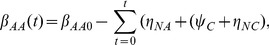
(13)

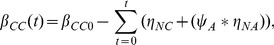
(14)

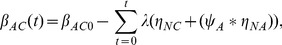
(15)

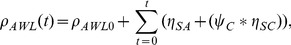
(16)

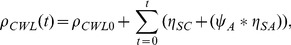
(17)

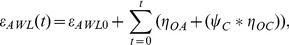
(18)

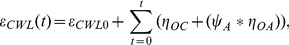
(19)


### Parameters Estimation

Model parameters were identified using existing US surveillance system data and research literature (Table S1). Stock variables were parameterized with 2009–2010 data from NHANES [37] to identify rates of normal, overweight and obesity in adults and children using current BMI and percentile guidelines. NHANES data in combination with recent research data were used to identify needed trends of flow between overweight and obese status (e.g., rates of dieting, exercise, and average time to lose weight). Finally, existing literature was used to provide coefficients for adult-to-adult, adult-to-child, and child-to-child social transmission. The details of parameter identification and estimation follow.

#### Engaging in Weight Loss Behaviors

Experts recommend both dietary changes and physical activity for obese and overweight children and adult weight loss [38], [39]. For clinically significant weight loss, recommendations include both a reduced calorie diet and a minimum of moderate-intensity physical activity for 250 minutes per week. For our parameters, we defined engaging in weight loss behaviors to apply to individuals who follow the recommended guidelines at a minimum, and calculated rates using data from the 2009–2010 NHANES dietary interview and the physical activity questionnaire [37]. Individuals who responded yes to following a “weight loss or low calorie diet” on the dietary interview were considered engaging in dieting for weight loss. Total minutes of weekly moderate and vigorous physical activity were calculated from the physical activity questionnaire by summing each respondent's typical number of days per week of moderate and vigorous recreational activity multiplied by time spent in minutes on a typical day in moderate and vigorous activity, respectively. Individuals who engaged in 250 minutes or more moderate and vigorous recreational activity per week were considered engaging in physical activity for weight loss. The proportion of individuals by stock variables (i.e., overweight adults, obese adults, overweight children, obese children) who were both dieting and doing physical activity for weight loss was calculated.

#### Obese and Overweight Adult Average Time to Return to Overweight and Normal Weight

These parameters were estimated using body measures from the 2009–2010 NHANES Anthropometry Examination [37] and a systematic review regarding expected weight loss for adults engaged in treatment programs involving diet and physical activity for weight loss [40]. The average amount of weight (in kg) obese and overweight adults needed to lose to transition to overweight or normal weight, respectively, was calculated using 2009–2010 NHANES data [37]. A review of overweight and obesity treatment indicated that adults can expect to lose an average of 5.5 kg in 12 months while engaged in behavioral modification for weight loss [40]. Thus, the average time overweight and obese adults needed to return to normal and overweight, respectively, was calculated by multiplying the average weight loss in proportion to the weight loss expected by the amount of time required for expected weight loss.

#### Obese and Overweight Child Average Time to Return to Overweight and Normal Weight

The average BMI decrease obese and overweight children need to transition to overweight or normal weight, respectively, was calculated using 2009–2010 NHANES Anthropometry Examination data [37]. The needed BMI decrease was calculated with respect to BMI-percentiles by age and gender with adjustment for time needed to decrease weight (one year for overweight to return to normal weight and two years for obese to return to overweight). A review of overweight and obesity treatment indicated that effective treatment interventions were shown to decrease children's BMI by 1.7 in one year while engaging in behavioral modification for weight loss [41]. Thus the average time overweight and obese children needed to return to normal and overweight, respectively, was calculated by multiplying the average BMI decrease needed in proportion to the BMI decrease expected by the amount of time required for expected BMI decrease.

#### Adult and Child Overweight Rate of Becoming Obese

The overweight adult and child rates of becoming obese were calculated using longitudinal data. The four year incidence of obesity of adult individuals in the Framingham longitudinal cohort study (data collected from 1979–2001) was found to be approximately 16% [42]. Thus, the rate for adults was calculated as: *γ_A_* = 0.16/(4 years *52 weeks per year) = 0.000769 week^−1^. The incidence of obesity in children who began as non-obese in a longitudinal study was found to be approximately 4.3% in 28 months [43]. Thus the rate for children was calculated as: *γ_C_* = 0.043/(28 months * 4.333 weeks per month) = 0.000354 week^−1^.

#### Social Transmission Rates

The adult-to-adult social transmission rate, β_AA_, was identified from numerical simulations reported from a study in Spain [29]. This value was used to define an appropriate range for sensitivity analysis for our research. In our experiments for the second objective, this value was assumed for the adult-to-adult social transmission rate and the child-to-child social transmission rate. The adult-to-child parameter was assumed to be 50% higher than the child-to-child social transmission rate due to evidence that child food intake is significantly higher in association with their parent's than peer's food intake [44]–[46].

### Simulation Experiments and Analysis

The first research objective was to conduct a sensitivity analysis on social transmission rates to determine their potential influence on childhood overweight and obesity prevalence. The second objective was to test alternative combinations of prevention and treatment intervention impacts at adult and child levels in order to determine where were the most impactful, based on varying degrees of adult intervention impact on children and vice versa. We used the combined childhood overweight and obesity prevalence as the decision criteria.

We defined a range of adult-to-child and child-to-child social transmission rates near reported values from the literature [29]. We conducted two-way sensitivity analyses using a set of 5 values each for adult-to-child and child-to-child social transmission rates in .0002 increments between 0.0011 and 0.0019. This range evaluated an adult-to-child social transmission rate that was between 0.58 to 1.73 times the child-to-child social transmission. A total of 25 total simulations using each potential combination of adult-to-child and child-to-child social transmission rates were run over a 10 year period. Baseline values of parameters were used and no interventions were applied.

We then defined 15 different combinations of adult and child obesity prevention and treatment interventions (Table 1), and tested a set of six scenarios that varied the adult obesity intervention impact factor at 25%, 50%, and 75%; and the child obesity intervention impact factor at 10% and 25% (Table 2). Quantification of these impact factors is limited, but research suggests a wide potential range for adult impact on children. For example, review studies note that a majority of research finds correlations between parents and child physical activity and food intake levels, but they range from weak to moderate levels [47], [48]. Knowledge regarding child impact on adults is also limited; however, a relatively weak influence is implied from awareness that child weight and behavioral interventions have minimal impact unless parents and home environments are also targeted [36], [49], [50]. Thus we chose to use scenarios to include a wide range of adult obesity intervention impact factors (i.e., 25%, 50%, and 75%), and a relatively low and smaller range of child obesity intervention impact factors (i.e., 10%, 25%), resulting in a total of 6 scenarios.

**Table 1:** Description of the Obesity Prevention and Treatment Intervention Alternatives.

	Alternatives	Description*
**AP**	Adult Obesity Prevention Interventions Only	Decrease in the adult-to-adult social transmission rate
**CP**	Child Obesity Prevention Interventions Only	Decrease in the child-to-child social transmission rate
**AT**	Adult Overweight & Obese Treatment Interventions Only	Decrease in overweight and obese adults engaging in weight loss behaviors
**CT**	Child Overweight & Obese Treatment Interventions Only	Increase in overweight and obese adults engaging in weight loss behaviors
**APCP**	Adult Obesity Prevention AND Child Obesity Prevention Interventions	Decrease in the adult-to-adult social transmission rate, and decrease in the child-to-child social transmission rate
**ATCT**	Adult and Child Overweight AND Obesity Treatment Interventions	Increase in overweight and obese adults and children engaging in weight loss behaviors
**ATCP**	Adult Overweight & Obesity Treatment AND Child Obesity Prevention Interventions	Increase in the overweight and obese adults engaging in weight loss behaviors and decrease in the child-to-child and adult-to-child social transmission rates
**APCT**	Adult Obesity Prevention AND Child Overweight & Obesity Treatment Interventions	Decrease in the adult-to-adult social transmission rate, and an increase in the overweight and obese children engaging in weight loss behaviors
**CPCT**	Child Obesity Prevention AND Child Overweight & Obese Treatment Interventions	Decrease in the child-to-child and adult-to-child social transmission rates, and an increase in the overweight and obese children engaging in weight loss behaviors
**APAT**	Adult Obesity Prevention AND Adult Overweight & Obese Treatment Interventions	Decrease in the adult-to-adult social transmission rate, and an increase in the overweight and obese adults engaging in weight loss behaviors
**APCPCT**	Adult Obesity Prevention AND Child Obesity Prevention AND Child Overweight & Obesity Treatment Interventions	Decrease in the adult-to-adult social transmission rate, and a decrease in the child-to-child and adult-to-child social transmission rates, and an increase in the overweight and obese children engaging in weight loss behaviors
**ATCPCT**	Adult Overweight & Obesity Treatment AND Child Obesity Prevention AND Child Overweight & Obesity Treatment Interventions	Increase in the overweight and obese adults and children engaging in weight loss behaviors, and a decrease in the child-to-child and adult-to-child social transmission rates
**APATCP**	Adult Obesity Prevention AND Adult Overweight & Obesity Treatment Interventions AND Child Obesity Prevention	Decrease in the adult-to-adult social transmission rate, and an increase in the overweight and obese adults engaging in weight loss behaviors, and decrease in the child-to-child and adult-to-child social transmission rates
**APATCT**	Adult Obesity Prevention AND Adult Overweight & Obesity Treatment Interventions AND Child Overweight & Obesity Treatment	Decrease in the adult-to-adult social transmission rate, and an increase in the overweight and obese adults engaging in weight loss behaviors, and an increase in the overweight and obese children
**ALL**	Adult Obesity Prevention AND Adult Overweight & Obese Treatment Interventions AND Child Obesity Prevention AND Child Overweight & Obese Treatment Interventions	Decreases in the adult-to-adult social transmission rate, and an increase in the overweight and obese adults and children engaging in weight loss behaviors, and a decrease in the child-to-child and adult-to-child social transmission rates

All interventions were modeled with a 50% continuous linear increase or decrease in designated parameters over the course of 10 years

**Table 2:** 3×2 Table of Defined Scenario Sets for Simulation Experiments.

	Adult Intervention Impact on Child (Ψ_A_, Adult Obesity Intervention Child Impact Factor)		
**Child Intervention Impact on Adult (Ψ_c_, Child Obesity Intervention Adult Impact Factor)**	***25%***	***50%***	***75%***
***10%***	Scenario 1	Scenario 2	Scenario 3
***25%***	Scenario 4	Scenario 5	Scenario 6

For each simulation, the targeted behavioral parameter was changed by 50% (social transmission rates were decreased and overweight and obese individuals engaging in weight loss behaviors was increased). The behavioral parameters were modeled to occur in a continuous linear change over a period of 10 years.

A lack of comprehensive data regarding weight loss behaviors and treatment and prevention interventions applied over the past several decades limited our ability to conduct formal statistical tests for model validation. As an alternative, we used a behavioral pattern testing approach [51] to compare our model simulations with US surveillance data trends. Our estimated and assumed baseline parameters produced an appropriate pattern and range of relative outcomes. From the early to mid-90s, US childhood overweight and obesity prevalence increased approximately 1.5 times [52]–[54] with recent evidence of potentially leveling rates [55]. Similarly, across our simulations (see results section), the childhood overweight and obesity prevalence increased from 1.45 to 1.97 across ten years, and in some scenarios a leveling to slight reduction is apparent.

## Results

**Figure 3:**
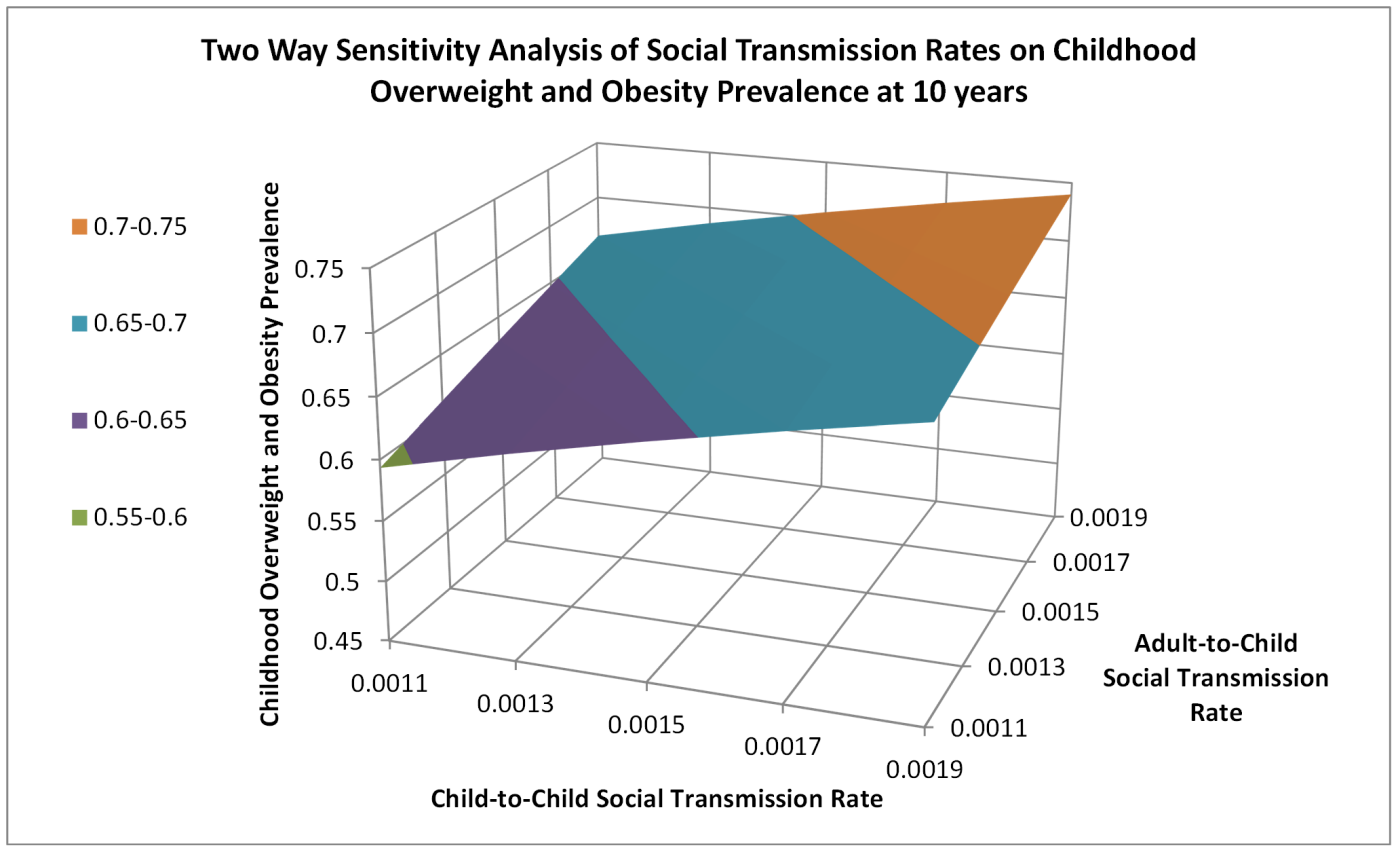
Two Way Sensitivity Analysis of Social Transmission Rates on Childhood Overweight and Obesity Prevalence at 10 years. This figure shows the results of the two-way sensitivity analysis of adult-to-child and child-to-child social transmission rates. The graph presents a three dimensional depiction of the childhood overweight and obesity prevalence at 10 years for each combination of adult-to-child and child-to-child social transmission rates tested. The chart indicates that the lowest childhood overweight and obesity prevalence is realized when both adult-to-child and child-to-child social transmission are at their lowest levels in each range. The change in overweight and obesity prevalence is greater across the adult-to-child than the child-to-child social transmission rate axis indicating slightly more sensitivity to the adult-to-child social transmission rate.

Figure 3 provides the results from the two-way sensitivity analysis. The prevalence rate was slightly more sensitive to the adult-to-child social transmission rate. For example, holding the converse rate constant, reducing the adult-to-child social transmission rate from .0019 to .0011 resulted in a 1.8% lower childhood overweight and obesity prevalence than the same reduction in the child-to-child social transmission rate.

**Figure 4:**
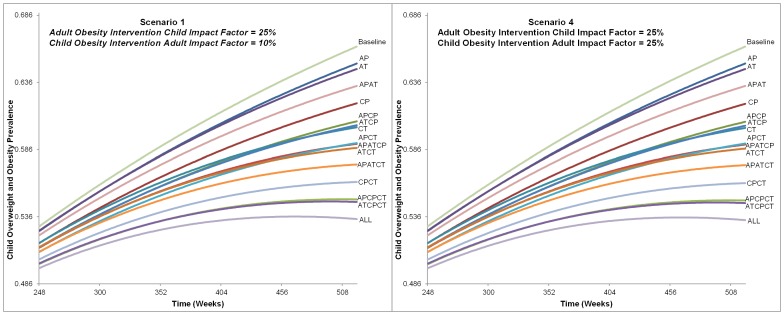
Alternatives Impact on Childhood Overweight and Obesity Prevalence from Scenarios 1 and 4. This figure shows the charts for each alternative from Scenario 1 and 4 influence on childhood overweight and obesity prevalence. The time frame charted is from 248 to 520 weeks. All alternatives are labeled and indicate that the ranking did not change between Scenario 1 or 4, nor was prevalence of each alternative greatly affected. The final childhood overweight and obesity prevalence ranges from approximately 53% with the intervention that included all intervention types and levels to 66% for baseline (no intervention).

**Figure 5:**
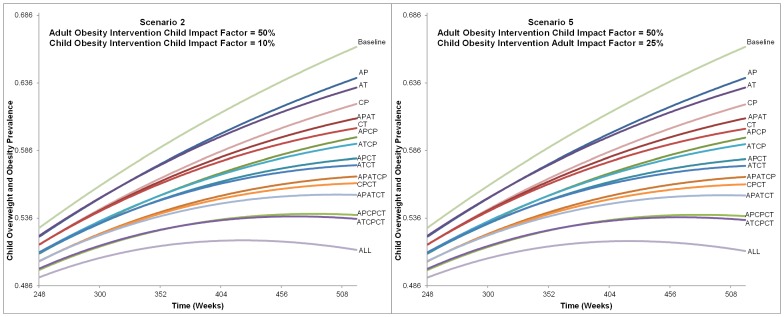
Alternatives Impact on Childhood Overweight and Obesity Prevalence from Scenarios 2 and 5. This figure shows the charts for each alternative from Scenario 2 and 5 influence on childhood overweight and obesity prevalence. The time frame charted is from 248 to 520 weeks. All alternatives are labeled and indicate that the ranking did not change between Scenario 2 or 5, nor was prevalence of each alternative greatly affected. The final childhood overweight and obesity prevalence ranges from approximately 51% with the intervention that included all intervention types and levels to 66% for baseline (no intervention).

**Figure 6:**
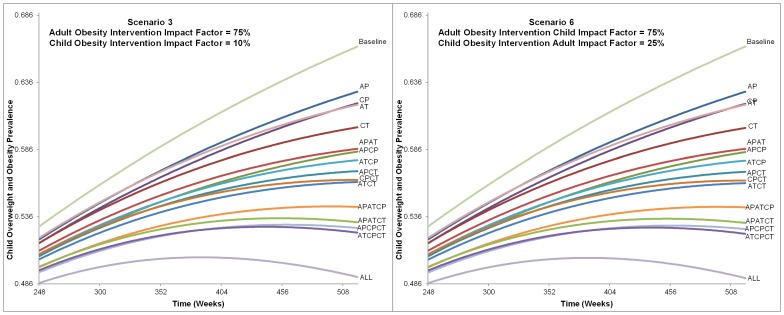
Alternatives Impact on Childhood Overweight and Obesity Prevalence from Scenarios 3 and 6. This figure shows the charts for each alternative from Scenario 3 and 6 influence on childhood overweight and obesity prevalence. The time frame charted is from 248 to 520 weeks. All alternatives are labeled and indicate that the ranking did not change between Scenario 3 or 6, nor was prevalence of each alternative greatly affected. The final childhood overweight and obesity prevalence ranges from approximately 49% with the intervention that included all intervention types and levels to 66% for baseline (no intervention).

Figures 4, 5, 6 provide the childhood overweight and obesity prevalence trends for each prevention and treatment intervention alternative from the six scenarios. Overall, many alternatives resulted in continued increase of childhood overweight and obesity prevalence, and only a few of the most comprehensive strategies (combining all or most treatment and prevention options) led to a downward trend by the end of the 10 years. Variation between the alternatives was not significantly apparent until after approximately 5 years (260 weeks).

As would be expected, the alternative that included all treatment and prevention options at both adult and child levels was the most impactful. Excluding this, the combination of adult treatment with child prevention and treatment interventions resulted in the lowest prevalence at the end of 10 years in all scenarios. Adult prevention alone resulted in the highest end prevalence at the end of 10 years. In each scenario, alternatives with treatment alone (targeted at either adults or children) reduced prevalence more than prevention alone (1.2–1.8% when targeted at children and 0.2 to 1.0% when targeted at adults).

Scenarios that compared different child intervention impact factors (10% versus 25%) with the same adult intervention impact factor did not result in large differences in childhood overweight and obesity prevalence. Comparing Scenarios 1 and 4; 2 and 5; and 3 and 6, the ranking of alternatives remained the same and the difference of the final prevalence was less than a tenth of a percent for each (Figures 4, 5, 6).

Conversely, the ranking of alternatives changed among scenarios that varied the adult intervention impact factor, and differences were seen in the final prevalence rates. Table 3 provides the ranking of alternatives and final childhood overweight and obesity prevalence for Scenarios 4–6. As the impact of adult interventions on children was increased, the rank of six alternatives that included adults became better (i.e., resulting in lower 10 year childhood overweight and obesity prevalence) than alternatives that only involved children. For example, in Scenario 4 (with an adult intervention impact factor of 25%), childhood treatment intervention only was ranked ninth best, better than adult prevention and treatment intervention combined (ranked thirteenth best). These alternatives' ranks changed in Scenario 6 (with adult intervention impact factor of 75%), where the childhood treatment intervention only was ranked twelfth, worse than adult prevention and treatment combined (ranked at eleventh). In Scenario 6, the adult treatment and child prevention alternative ranked as ninth.

**Table 3:** Ranking and Final Childhood Overweight and Obesity Prevalence for Scenarios 4–6.

	Adult-to-child Impact Factor*					
	25% (Scenario 4)		50% (Scenario 5)		75% (Scenario 6)	
	Alternative	Final Childhood Overweight and Obesity Prevalence	Alternative	Final Childhood Overweight and Obesity Prevalence	Alternative	Final Childhood Overweight and Obesity Prevalence
Highest Final Childhood Overweight and Obesity Prevalence	AP	65.01%	AP	63.97%	AP	62.91%
	AT	64.60%	AT	63.26%	CP	62.01%
	APAT	63.34%	CP	62.01%	AT	61.94%
	CP	62.01%	APAT	60.98%	CT	60.21%
	APCP	60.67%	CT	60.21%	APAT	58.65%
	ATCP	60.37%	APCP	59.55%	APCP	58.41%
	CT	60.21%	ATCP	59.07%	ATCP	57.78%
	APATCP	59.05%	APCT	57.97%	APCT	56.95%
	APCT	58.97%	ATCT	57.48%	ATCT	56.30%
	ATCT	58.67%	APATCP	56.66%	CPCT	56.11%
	APATCT	57.45%	CPCT	56.11%	APATCP	54.30%
	CPCT	56.11%	APATCT	55.28%	APATCT	53.14%
	APCPCT	54.81%	APCPCT	53.75%	APCPCT	52.68%
	ATCPCT	54.62%	ATCPCT	53.47%	ATCPCT	52.33%
Lowest Final Childhood Overweight and Obesity Prevalence	APATCPCT	53.35%	APATCPCT	51.17%	APATCPCT	49.03%

Child to Adult Impact Factor is 25% for all Scenarios.

Alternatives with greater numbers of intervention types (prevention and treatment at adult and child levels) did not directly correspond to better ranking. For example, in Scenarios 1 and 4 five alternatives with fewer intervention types had better rankings than alternatives with more intervention types. For example, including two intervention types (child prevention and child treatment) resulted in a better ranking than several alternatives that included three intervention types but were more adult focused (i.e., adult prevention and treatment combined with child prevention only or adult prevention and treatment combined with child treatment only). Conversely, in Scenarios 3 and 6 any alternative that included three intervention types (regardless of adult or child focus) was better ranked than any with only two.

## Discussion and Conclusions

This research provides new insight that has implications on future policies and decision-making regarding prevention versus treatment intervention combinations and adult versus peer levers of social influence. Childhood obesity prevalence may be more sensitive to changes in adult-to-child social transmission rates compared to child-to-child rates. Similar to previous modeling research [29], our experiments found that combinations of prevention and treatment generally have greater impact than either alone. However, the additional complexity of adult and child influences and social transmission resulted in changes to an alternative's impact depending on varying influence of adult and child interventions on each other.

The two-way sensitivity analyses revealed that childhood obesity and overweight prevalence is sensitive to changes in social transmission rates from both adult and peer levels. Using current surveillance data from the US for baseline values and no interventions, changes to the adult-to-child transmission rate had slightly greater impact than child-to-child on childhood overweight and obesity. Current research strongly suggests the presence of social influences on obesity and obesity-related health behaviors [9]. However, the quantification of social transmission is limited in current research. Compared to infectious disease, the complexities of issues such as longer exposure timeframes and nuanced social protective and risk factors make exploration of such quantification more difficult. Research that attempts to intervene on social transmission at adult-to-child and child-to-child levels may help to elucidate the mechanisms and improve the target within interventions.

Our findings also indicate that the combination of prevention and treatment interventions may need to consider the social transmission context for optimum impact. Within any of our scenarios, alternatives that included treatment intervention impact (especially targeted at child levels) versus a prevention intervention impact, resulted in lower childhood overweight and obesity prevalence after 10 years. Santonja et al [29] found that for adults in Spain, prevention alone strategies resulted in greater reductions of overweight and obesity. The difference in our findings is possibly due to the higher initial prevalence of overweight and obesity found in the US and used for our model parameters. Determining priorities regarding prevention or treatment interventions for obesity and chronic diseases is a source of ongoing debate, though, most concede a blend of both approaches are needed [56]–[60]. Our research does not minimize the importance or potential of obesity prevention interventions, but challenges us to consider how a society with high prevalence of overweight and obesity and noted obesogenic socio-cultural environments [61] might respond to prevention interventions that simply seek to educate and change attitudes about healthy lifestyles.

Furthermore, evidence that combinations of prevention and treatment interventions are most influential encourage thoughtful consideration of how both strategies should address mechanisms of social transmission. The role of social-cultural environments is evident in multilevel and systems-oriented models for obesity intervention [7], [61] and can be useful to conceptualize and define targets for both prevention and treatment interventions at population-levels. For example, interventions should consider how to target social norms regarding the desire and advocacy for environments that support healthy behaviors for both prevention and weight loss.

Our research also tested the potential for interventions to act through targeted mechanisms of adult influence on children and vice versa (e.g., actively engaging individuals to support and encourage others in their social environment in healthy behaviors).The results indicated childhood overweight and obesity prevalence is sensitive to adult influence. The ranking of alternative interventions at child and adult levels changed based on the degree of influence adult interventions had on children. Intervention combinations that focus more heavily on adults may result in greater reductions in childhood obesity than those that target children only if adult interventions have higher residual impact on children. Targeting children has been noted as advantageous due to issues such as political expediency [62] and relative ease of shifting behavior [63]; however, our results indicate that if effective interventions are available, targeting adults may be more efficient. A recent intervention study found that a parent-only intervention resulted in equal impact on child weight loss as compared to those that included both parents and children [64], and that parent weight loss was slightly better for the parent only intervention group. Research should seek to expand and strengthen this type of intervention.

The results of our research indicate the potential for such methodologies to aid in intervention planning and finite resource allocation by determining the potential impact of different intervention combinations. It is noted that public health policy makers can be overwhelmed by the complicated task of using data, evidence, reviews and summaries to determine best practices [65]. Our model provides evidence about the impact of different combinations, which could be combined with decision making models that includes factors such as adult influence and cost, to assist with resource allocation decisions.

This research does have its limitations. The current model and research is deterministic and was not built for predictive purposes. It does not allow testing for statistical significance between the intervention combinations. Another key constraint of the model is the assumption of homogenous mixing. Research has indicated that social transmission is likely to occur through clustering effects and spreads differently through various types of social ties [8], [66]. Regardless, the model does allow consideration of patterns and outcomes that point to potential gaps in current research and new hypotheses about plausible intervention levers.

System dynamics modeling provides a tool that can strengthen the connections between generation, synthesis, and translation of evidence. Our results highlight areas of research that could provide beneficial information to inform future modeling and enhance decision making. Better quantification of the relative impact of adult-to-adult, adult-to-child, and child-to-child social influences in terms of transference of both unhealthy and healthy behaviors would strengthen our ability to answer questions regarding optimum combinations of interventions. However, in the absence of such information the model can still provide valuable insight into potential patterns and trends.

Future research can use and expand this model to answer additional research questions. This model established the core structure for modeling the child and adult dynamic social transmission of unhealthy obesity-related behavior, but future work is needed to expand the model boundaries to include elements of intervention implementation (i.e., intervention resources, cost, demand, and supply) (Figure S1). The model should also be considered in combination with agent-based models to explore the influence of networks and clustering in different population structures. Finally, future studies should use this model to explore important leverage points in order to harness impact and target the different combinations of interventions dynamically. For example, the rate of change in childhood overweight and obesity prevalence may be greater with certain alternative policy combinations. Thus, points of inflection can be identified to improve our understanding when and how alternatives should be planned and implemented through time.

## Supporting Information

Table S1 **Definition, Description, and Values of Model Parameters.** This table provides more detailed description of the model parameters used for the current study.
(DOCX)

Figure S1 **Expanded Causal Loop Diagram of Social Transmission of Overweight and Obesity for Future Research.** This causal loop diagram expands the model used for the current research. The same system elements are included in order to map the adult and child social transmission of overweight and obesity, but new elements and connections are added that can be used to explore the influence of resource availability, intervention implementation cost, supply of interventions, and demand of interventions. The element of resources is now shown with four arrows to adult overweight and obesity treatment, adult prevention, child overweight and obesity treatment, and child prevention interventions. A plus sign is indicated for each of these arrows indicating that an increase of resources will increase intervention implementation. Each respective intervention implementation has an arrow to the appropriate impact (e.g., adult prevention intervention implementation will increase adult prevention intervention impact). The normal weight populations (child and adult) are shown with an arrow and plus sign to link to demand for prevention interventions. Likewise, the overweight and obese populations are shown with arrows and plus signs to link to demand for treatment interventions. Finally, demand is shown with an arrow and plus sign to intervention implementation indicating a positive relationship.
(TIF)
