# Correction: Modeling Social Transmission Dynamics of Unhealthy Behaviors for Evaluating Prevention and Treatment Interventions on Childhood Obesity

**DOI:** 10.1371/journal.pone.0097204

**Published:** 2014-05-02

**Authors:** The PLOS ONE Staff

The captions in Figures 5 and 6 are incorrect. Please see the corrected figures below.

**Figure 5:**
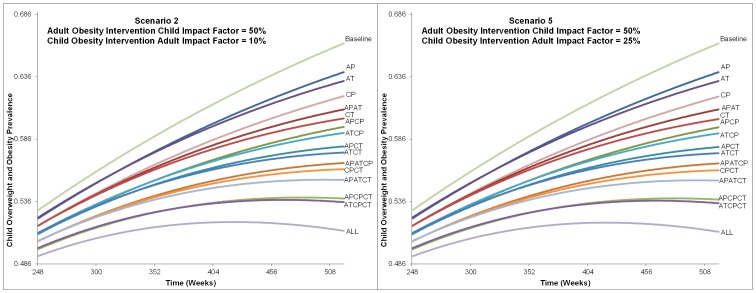
Alternatives Impact on Childhood Overweight and Obesity Prevalence from Scenarios 2 and 5. This figure shows the charts for each alternative from Scenario 2 and 5 influence on childhood overweight and obesity prevalence. The time frame charted is from 248 to 520 weeks. All alternatives are labeled and indicate that the ranking did not change between Scenario 2 or 5, nor was prevalence of each alternative greatly affected. The final childhood overweight and obesity prevalence ranges from approximately 51% with the intervention that included all intervention types and levels to 66% for baseline (no intervention).

**Figure 6:**
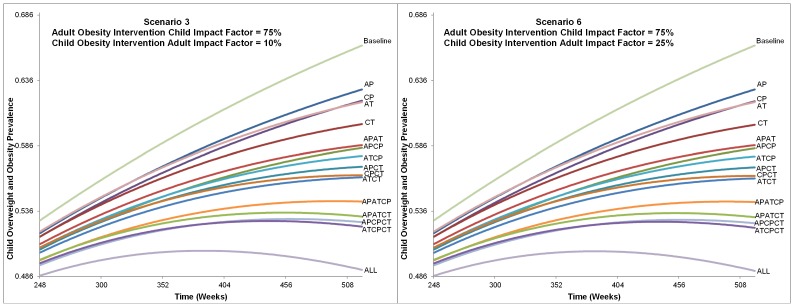
Alternatives Impact on Childhood Overweight and Obesity Prevalence from Scenarios 3 and 6. This figure shows the charts for each alternative from Scenario 3 and 6 influence on childhood overweight and obesity prevalence. The time frame charted is from 248 to 520 weeks. All alternatives are labeled and indicate that the ranking did not change between Scenario 3 or 6, nor was prevalence of each alternative greatly affected. The final childhood overweight and obesity prevalence ranges from approximately 49% with the intervention that included all intervention types and levels to 66% for baseline (no intervention).
