# Supplementary material for: Modeling Social Transmission Dynamics of Unhealthy Behaviors for Evaluating Prevention and Treatment Interventions on Childhood Obesity
Source: PLoS One. 2013 Dec 17;8(12):e82887. doi: 10.1371/journal.pone.0082887 (PMC3866177; doi:10.1371/journal.pone.0082887)
Supplement: Table S1 — Definition, Description, and Values of Model Parameters. This table provides more detailed description of the model parameters used for the current study. (DOCX) [file pone.0082887.s001.docx]

**Table S1. Definition, Description, and Values of Model Parameters**

| **Stock Variables** | **Definition** | **Data** | **Data Source** |
| --- | --- | --- | --- |
| N_A_, Normal Weight Adults | Proportion of individuals ages 20 and older with BMI < 25 | 30.8% | Estimated from 2009-2010 NHANES as reported in Flegal et al [67] |
| S_A_, Overweight Adults | Proportion of individuals 20 and older with BMI ≥ 25 and < 30 | 33.3% | Estimated from 2009-2010 NHANES as reported in Flegal et al [67] |
| O_A_, Obese Adults | Proportion of individuals 20 and older with BMI ≥ 30 | 35.9% | Estimated from 2009-2010 NHANES as reported in Flegal et al [67] |
| N_c_, Normal Weight Children | Proportion of children ages 12-19 < 85^th^ Percentile on BMI-for age and gender growth charts | 66.4% | Estimated from 2009-2010 NHANES as reported in Ogden et al [5] |
| S_c_, Overweight Children | Proportion of children ages 12-19 ≥ 85^th^  and < 95^th^ Percentile on BMI-for age and gender growth charts | 15.2% | Estimated from 2009-2010 NHANES as reported in Ogden et al [5] |
| O_c_, Obese Children | Proportion of children ages 12-19 ≥ 95^th^ Percentile on BMI-for age and gender growth charts | 18.4% | Estimated from 2009-2010 NHANES as reported in Ogden et al [5] |
| **Constants** |  |  |  |
| ρ_AWL_, Overweight Adults Engaging in Weight Loss Behaviors | Proportion of overweight adults who indicate both being on a special weight loss/low calorie diet AND getting more than 250 minutes of moderate or vigorous recreational activity per week | 5.60% | Estimated from 2009-2010 NHANES [40] (see parameters estimation) |
| ε_AWL_, Obese Adults Engaging in Weight Loss Behaviors | Proportion of obese adults who indicate both being on a special weight loss/low calorie diet AND getting more than 250 minutes of vigorous activity per week | 11.04% | Estimated from 2009-2010 NHANES [40] (see parameters estimation) |
| *p_SA_*, Overweight Adult average time to return to normal weight | 1/ *p_SA_* is the average time for an overweight adult following behavioral modifications needs to return to normal weight | 1/65 weeks^-1^ | Estimated using 2009-2010 NHANES and USPSTF systematic review of adult obesity treatment effects [40,43] (see parameters estimation) |
| *p_oA ,_* Obese Adult average time to return to overweight | 1/ *p_oA_* is the average time an obese adult following behavioral modifications needs to return to overweight | 1/153 weeks^-1^ | Estimated using 2009-2010 NHANES and USPSTF review of adult obesity treatment effects [40,43] (see parameters estimation) |
| ρ_CWL_, Overweight Child Engaging in Weight Loss Behaviors | Proportion of overweight children who indicate both being on a special weight loss/low calorie diet AND getting more than 250 minutes of moderate or vigorous recreational activity per week | 2.23% | Estimated from 2009-2010 NHANES [40] (see parameters estimation) |
| ε_CWL_, Obese Child Engaging in Weight Loss Behaviors | Proportion of obese children who indicate both being on a special weight loss/low calorie diet AND getting more than 250 minutes of moderate or vigorous recreational activity per week | 6.64% | Estimated from 2009-2010 NHANES [40] (see parameters estimation) |
| *p_SC_* , Overweight child average time to return to normal weight | 1/*p_SC_* is the average time for an overweight child following behavioral modifications needs to return to overweight | 1/40 weeks^-1^ | Estimated using 2009-2010 NHANES and USPSTF review of childhood obesity treatment effects [40,44] (see parameters estimation) |
| *p_oC ,_* Obese child average time to return to normal weight | 1/ *p_oC_*  is the average time for an obese child following behavioral modifications needs to return to overweight | 1/132 weeks^-1^ | Estimated using 2009-2010 NHANES and USPSTF review of childhood obesity treatment effects (see parameters estimation) |
| *ϒ_A ,_* Overweight Adult Rate of Becoming Obese | Rate at which overweight adults become obese | .000769% weeks^-1^ | Estimated from obesity incidence rate from the Framingham longitudinal study (1979-2001) [45] (see parameters estimation) |
| *ϒ_C ,_* Overweight Child Rate of Becoming Obese | Rate at which overweight children become obese | .000354% weeks^-1^ | Estimated for obesity incidence rate from obesity incidence rate from a child weight longitudinal study (2006-2009) [46] (see parameters estimation) |
| β_AA_, Adult-to-adult Social Transmission Rate | Social transmission rate of adoption of unhealthy lifestyles from overweight and obese adults to normal weight adults | .001121% weeks^-1^ | Value used from previous research - Santonja et al [29] |
| β_CC_, Child-to-child Social Transmission Rate | Social transmission rate of adoption of unhealthy lifestyles from overweight and obese children to normal weight children | .001121% weeks^-1^ | Value set equal to adult-to-adult transmission rate from Santonja et al [29] |
| β_AC_, Adult-to-child Social Transmission Rate | Social transmission rate of adoption of unhealthy lifestyles from overweight and obese adults to normal weight children | .001682% weeks^-1^ | Value assumed at 150% of the child-to-child transmission rate |
| **Intervention Parameters** |  |  |  |
| ɳ_oc,_ childhood obesity treatment intervention impact | Rate of impact on the proportion of obese children engaging in weight loss behaviors | 2.14423 e-5 % weeks^-1^ | Value modeled in order to achieve a 50% increase over 10 years |
| ɳ_sc_, childhood overweight treatment intervention impact | Rate of impact on the proportion of overweight children engaging in weight loss behaviors | 6.38462 e-5 % weeks^-1^ | Value modeled in order to achieve a 50% increase over 10 years |
| ɳ_NC_, childhood prevention treatment intervention impact | Rate of impact on the adult-to-child and child-to-child social transmission rates | 1.07788 e-6 % weeks^-1^ | Value modeled in order to achieve a 50% decrease over 10 years |
| ɳ_OA_, adult obesity treatment intervention impact | Rate of impact on the proportion of obese adults engaging in weight loss behaviors | 1.06154 e-4 % weeks^-1^ | Value modeled in order to achieve a 50% increase over 10 years |
| ɳ_SA_, adult overweight treatment intervention impact | Rate of impact on the proportion of overweight adults engaging in weight loss behaviors | 5.38462 e-5 % weeks^-1^ | Value modeled in order to achieve a 50% increase over 10 years |
| ɳ_NA_, adult obesity prevention treatment intervention impact | Rate of impact on the adult-to-adult social transmission rates | 1.07788 e-6 % weeks^-1^ | Value modeled in order to achieve a 50% decrease over 10 years |
| $\lambda$, discount factor | Factor to account for resistance of adult-to-child social transmission to respond to child prevention interventions | 0.5 (dimensionless) | Value assumed to be 50% of total childhood obesity prevention impact |
